# Supplementary material for: Contribution of boundary non-stoichiometry to the lower-temperature plasticity in high-pressure sintered boron carbide
Source: Nat Commun. 2023 Aug 21;14:4889. doi: 10.1038/s41467-023-40581-7 (PMC10442403; doi:10.1038/s41467-023-40581-7)
Supplement: Supplementary file 1 — Supplementary Information [file 41467_2023_40581_MOESM1_ESM.pdf]

Supplementary Information

Contribution of boundary non-stoichiometry to the lower-temperature plasticity in high-pressure sintered boron carbide

Haiyue Xu [1 #], Wei Ji [1,2 # *], Jiawei Jiang [2], Junliang Liu [2], Hao Wang [1], Fan Zhang [1,3], Ruohan Yu [4], Bingtian Tu [1], Jinyong Zhang [1], Ji Zou [1], Weimin Wang [1], Jinsong Wu [1,4], Zhengyi Fu [1 *]

1. State Key Laboratory of Advanced Technology for Materials Synthesis and Processing, Wuhan University of Technology, Wuhan 430070, China

2. Department of Materials, University of Oxford, Oxford OX1 3PH, UK

3. Hubei Longzhong Laboratory, Wuhan University of Technology Xiangyang Demonstration Zone, Xiangyang 441000, China

4. Nanostructure Research Centre, Wuhan University of Technology, Wuhan 430070, China

*Corresponding author: jiwei@whut.edu.cn; zyfu@whut.edu.cn

[#]These authors contributed equally: Haiyue Xu, Wei Ji

*These authors jointly supervised this work: Wei Ji, Zhengyi Fu

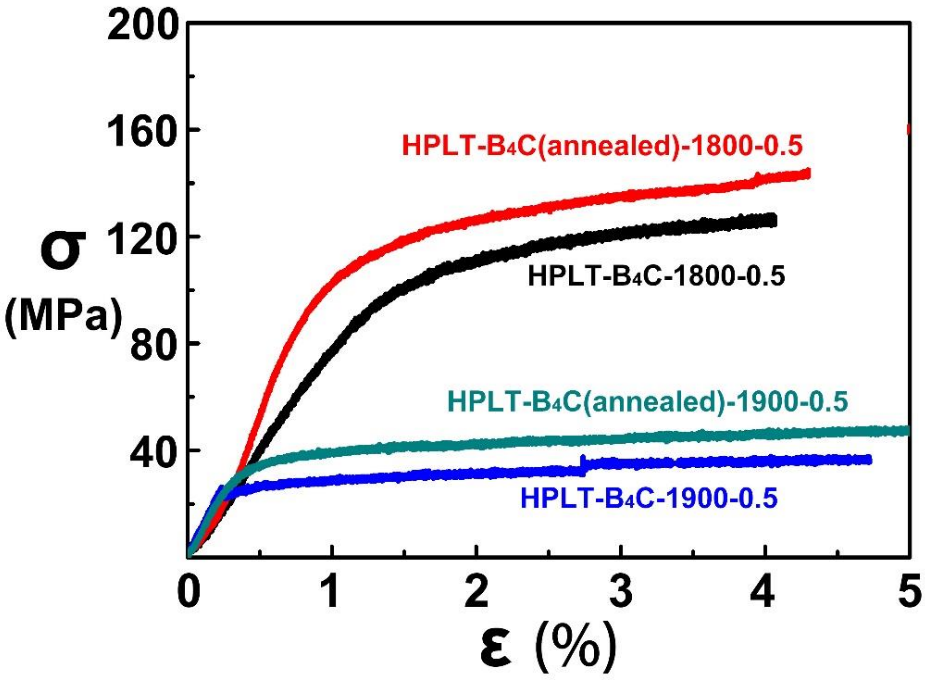

Supplementary Figure 1. Effects of annealing on the high-temperature plasticity of HPLT-B_4C. The stress-strain curves include HPLT-B_4C-1800-0.5, HPLT-A-B_4C-1800-0.5, HPLT-B_4C-1900-0.5 and HPLT-A-B_4C-1900-0.5, which were listed in Supplementary Table 1. Source data are provided as a Source Data file.

To determine the effect of thermal activated stress relief on the hardening mechanism of high-temperature yield stress and plastic deformation temperature, the flexible behaviors at 1800 °C and 1900 °C of HPLT-B_4C after annealing at 1900 °C are illustrated in Supplementary Fig. 1. The strength values of HPLT-B_4C-1800-0.5, HPLT-A-B_4C-1800-0.5, HPLT-B_4C-1900-0.5 and HPLT-A-B_4C-1900-0.5 were 120 MPa, 140 MPa, 35 MPa and 45 MPa, respectively. The results indicated that although annealing could raise strength value at high temperatures, it had almost no effect on the plastic deformation temperature.

Supplementary Table 1. Summary of Testing Conditions of B_4C ceramics with or without annealing

Samples	Sintering Temperatures (°C)	Sintering Pressures (MPa)	Annealing Temperatures (°C)	Annealing Time (min)	Test Temperatures (°C)	Crosshead Speed (mm/min)
**HPLT-A-B_4C-1800-0.5**	1800	80	1900	30	1800	0.5
**HPLT-A-B_4C-1900-0.5**	1800	80	1900	30	1900	0.5
**HPLT-B_4C-1800-0.5**	1800	80	—	—	1800	0.5
**HPLT-B_4C-1900-0.5**	1800	80	—	—	1900	0.5

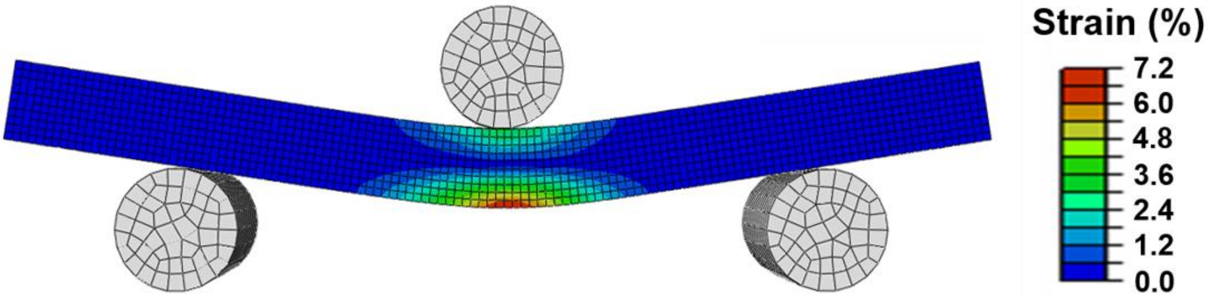

Supplementary Figure 2. Typical strain diagram of B_4C ceramics during high-temperature flexural test from finite element analysis.

B_4C ceramics samples exhibited plastic properties at high temperatures similar to metals. The B_4C test bar was simulated with the plastic mechanical model to determine the strain distribution during the high-temperature plastic deformation process. The finite element analysis proved that the main strain fields were located at outer edges of the central region, followed by inner edges of the central region. The ends of the test bar did not show obvious strain.

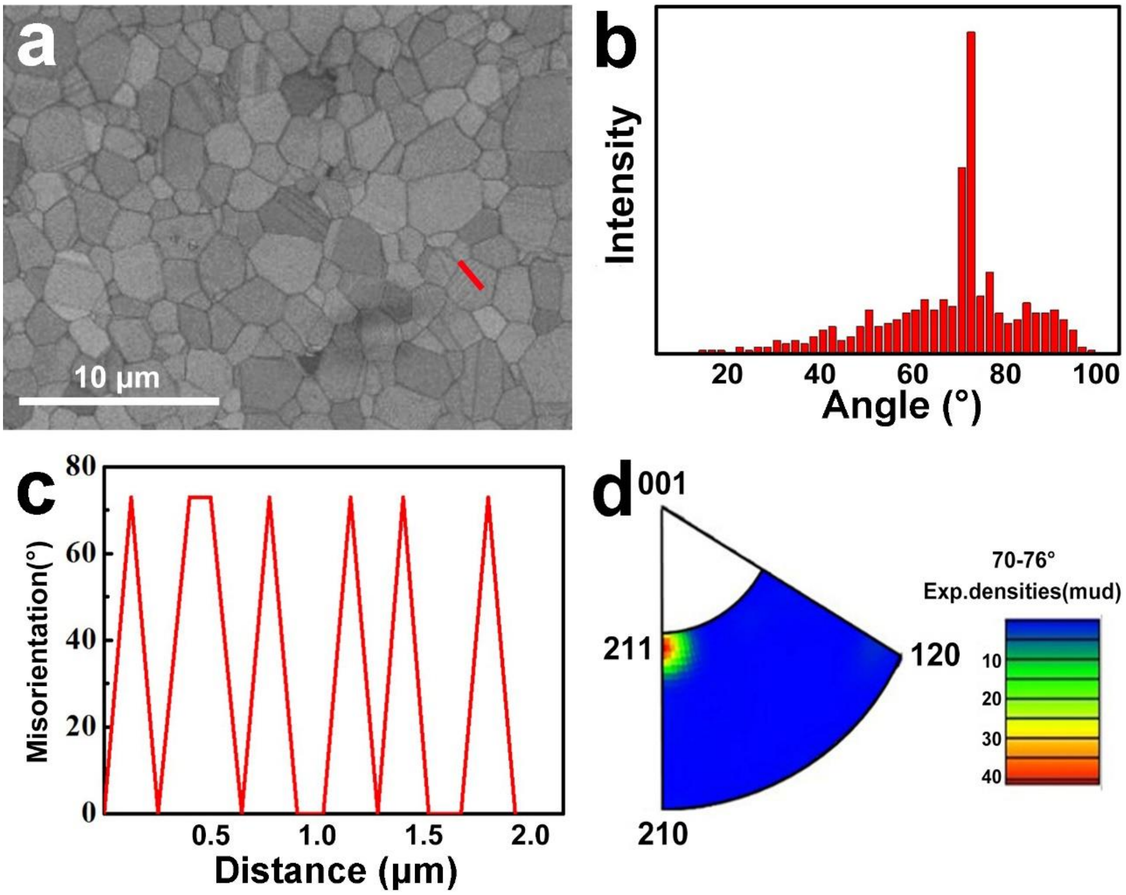

Supplementary Figure 3. Electron back scattered diffraction (EBSD) results of the typical morphology for press-assisted sintered B_4C before high-temperature flexural test. (a) Band contrast micrograph. **(b)** Misorientation distribution. **(c)** Measured misorientation across the interfaces along the red line. **(d)** Misorientation axes in crystal coordinates in the range of 70-76°. Source data are provided as a Source Data file.

The misorientation distribution of the original fully dense B_4C (Supplementary Fig. 3(b)) revealed that low-angle grain boundaries hardly existed. Consequently, there are rare lattice distortion-induced geometrically necessary dislocations. Futhermore, B_4C ceramics contained a high-density of 73°<211> twins (Supplementary Figs. 3(c) and (d)).

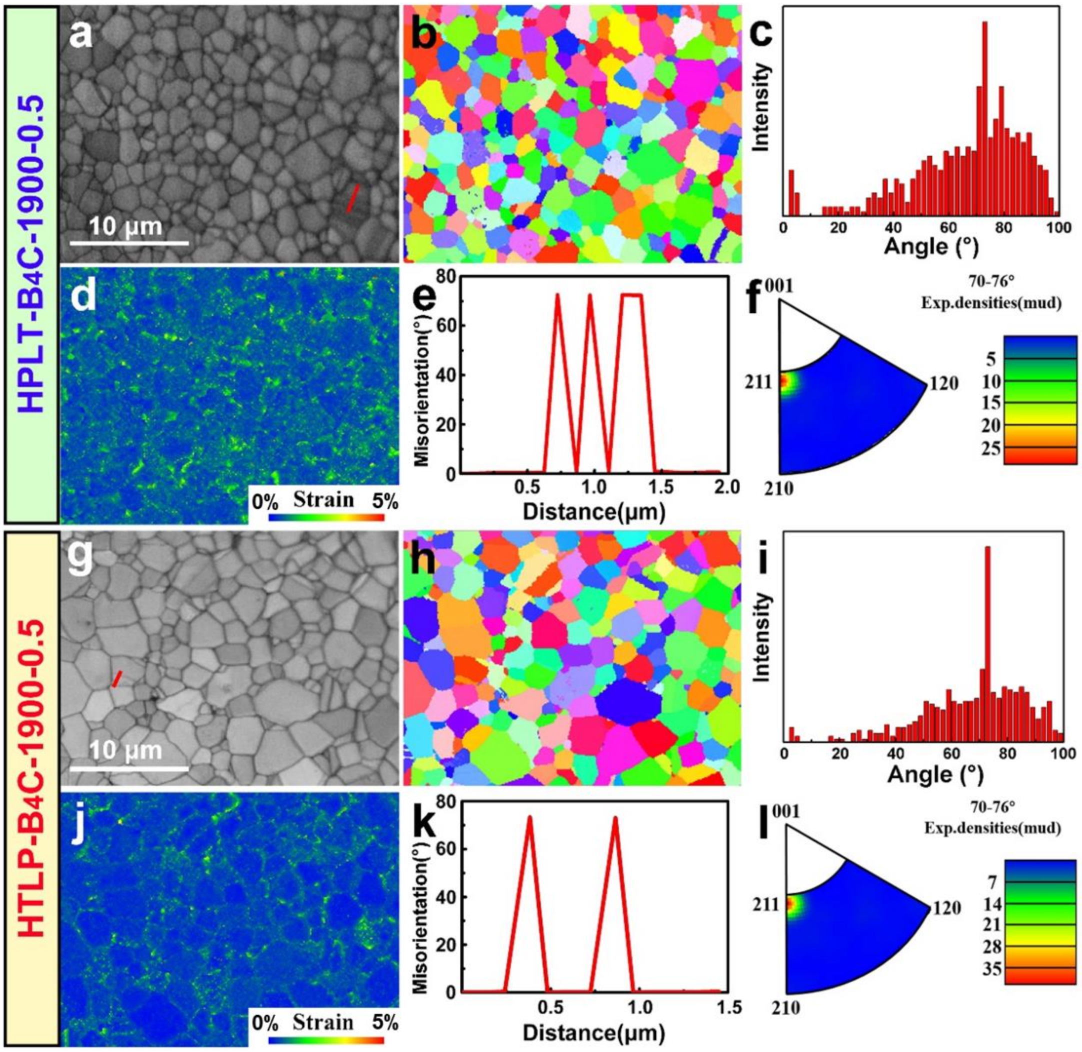

Supplementary Figure 4. EBSD results indicating the microstructures of B_4C ceramics after high-temperature flexural test. (a-f) HPLT-B_4C-1700-0.5 and **(g-l)** LPHT-B_4C-1900-0.5. **(a, g)** Band contrast micrograph. **(b, f)** Representative orientation map. **(c, i)** Misorientation distribution. **(d, j)** Local misorientation mapping analysis. **(e, k)** Measured misorientation across the interfaces along the red line in band contrast images. **(f, i)** Misorientation axes in crystal coordinates in the range of 70-76° of the tensile region. Source data are provided as a Source Data file.

The EBSD results in Supplementary Fig. 4 shows that the grains of both deformed samples were still equiaxed without texture evolution and completely different from the morphology of metals after plastic deformation. The misorientation characterizations (Supplementary Figs. 4(c) and 4(i)) illustrated the formation of low-angle grain boundaries. Supplementary Figs. 4(e) and 4(k) depict the misorientations and twin angles along the red lines in Supplementary Figs. 4(a) and 4(g). Supplementary Figs.

4(f) and 4(l) show the misorientation axes of boundaries close to the misorientations of 73° indicated for the twins. The results demonstrate that the twinning is 73°<211> in both HPLT-B_4C and LPHT-B_4C ceramics and that their widths were typically 300 nm. The results were compared with the EBSD results before high-temperature strength test (Supplementary Fig. 3). The twinning system and width of twins in B_4C ceramics after high-temperature deformation remained constant, suggesting that the twin multiplication was not the controlling mechanism for high-temperature plastic deformation of B_4C ceramics.

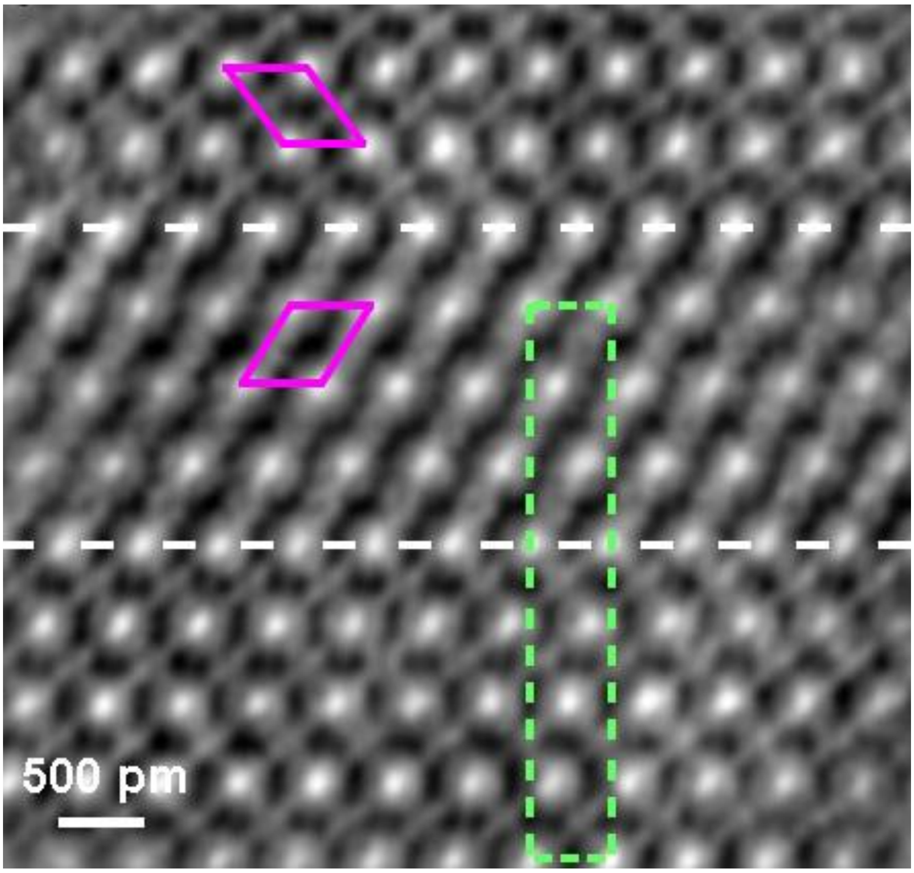

Supplementary Figure 5. ACTEM image of intragranular asymmetric nanotwins in HPLT-B_4C sample. It was reported that B_4C with a B:C ratio of 4:1 was found to undergo both symmetric and asymmetric twinning, whereas boron-rich carbides only underwent symmetric twinning[1-2]. The observation suggests that the B:C ratio of the as-sintered B_4C ceramics was 4:1.

The results indicate that nano-twins have been induced by the high-pressure sintering technology. In addition, this asymmetric feature can be elucidated with the close periodic units (dotted green rectangles) drawn on both sides of the twin interface. The top and bottom lattices show obviously different atomic-column periodicities normal to the twin, namely, three- and four-column periodicity.

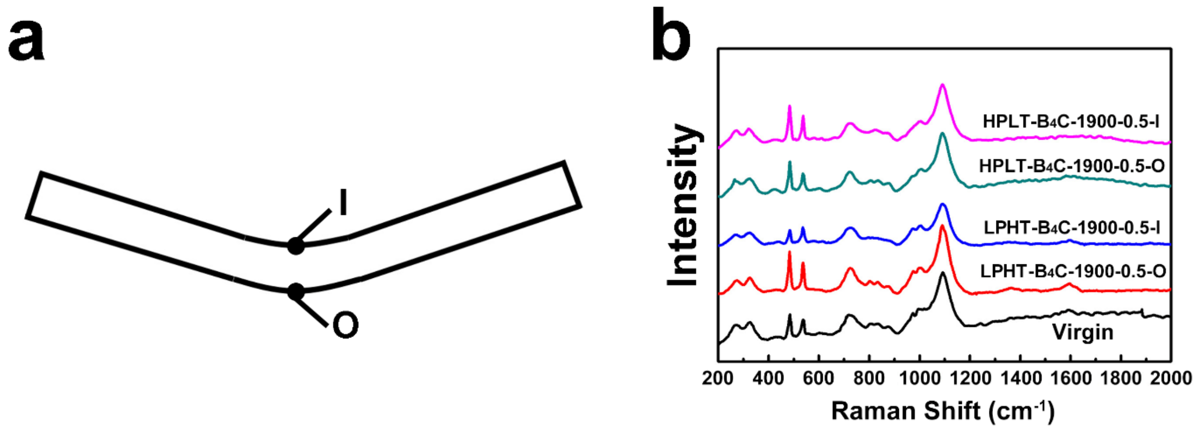

Supplementary Figure 6. Raman spectra of different locations in the bended B_4C ceramics. (a) The schematic of a bended sample. The concentrated deformation zones were located at inside (marked as I) and outside (marked as O). **(b)** Raman spectra of B_4C ceramics (HPLT-B_4C-1900-0.5 and LPHT-B_4C-1900-0.5) at the corresponding loctions after high-temperature flexural test compared to a control sample without residual stress (Virgin). The results comfirmed that no phase change occurred during the tests. Source data are provided as a Source Data file.

Both HPLT-B_4C and LPHT-B_4C exhibited obvious plasticity at 1900 °C. The potential phase transition and residual stress in the high-temperature plastic deformation were investigated by Raman spectroscopy. Based on finite element analyses of the flexible deformation, the severe deformation zones were located at inside (I) and outside (O) in Supplementary Fig. 6(a). Parsard [3] correlated the frequency shift of a Raman peak at about 1090 cm^{-1} with the average value of residual stress:

$$\sigma_{res} = k\Delta w \quad , \tag{1}$$

where σ_{res} is the residual stress; k is the piezospectroscopic constant, 364 MPa/cm; Δw is the change in the frequency of a peak between virgin B_4C ceramics and other samples with internal stresss. The virgin B_4C ceramics was the LPHT-B_4C obtained after annealing at 1900 °C for 30 min with a heating rate of 10 °C/min. The large plastic deformation areas of the samples corresponding to I and O after high-temperature flexible test were selected as the test positions.

Supplementary Table 2 Raman peaks, peak shifts and residual stresses of HPLT-B_4C-1900-0.5 and LPHT-B_4C-1900-0.5 after high-temperature plastic deformation

Samples	Raman peaks (cm^{-1})	Peak shifts (cm^{-1})	σ_{res} (MPa)
Virgin	1092.8	0	0
HPLT-B_4C-1900-0.5-O	1091.9	-0.9	327.6
HPLT-B_4C-1900-0.5-I	1092.1	-0.7	254.8
LPHT-B_4C-1900-0.5-O	1092.0	-0.8	291.2
LPHT-B_4C-1900-0.5-I	1092.4	-0.4	145.6

The residual stresses of the HPLT-B_4C-1900-0.5 and LPHT-B_4C-1900-0.5 calculated with Eq. (1) are listed in Supplementary Table 2. Neither amorphized zones nor amorphous peaks[4] were detected in microstructure characterization or Raman spectra analysis. The solid state amorphization reaction was not activated under the load-induced plastic strain at a high temperature (~1900 °C). The result was completely different from that at ambient temperature[5]. The low residual stress and the absence of amorphous phase indicated that B_4C ceramics had the characteristics of stability in the high-temperature plastic deformation.

Supplementary References

[1] Goddard, W. A. I., Hemker, K. J., Xie, K. Y. Atomic-Level Understanding of "Asymmetric Twins" in Boron Carbide. *Phys. Rev. Lett.* **115**, 175501 (2015).

[2] Awasthi, A., Subhash, G. Deformation behavior and amorphization in icosahedral boron-rich ceramics. *Prog. Mater. Sci.* **112**, 100664 (2020).

[3] Parsard, G., Subhash, G., Jannotti, P. Amorphization-induced volume change and residual stresses in boron carbide, *J. Am. Ceram. Soc.* **101**, 2606-2615 (2018).

[4] Guo, D. Grain boundary sliding and amorphization are responsible for the reverse Hall-Petch relation in superhard nanocrystalline boron carbide. *Phys. Rev. Lett.* **121**, 145504 (2018).

[5] Yan, X. Q., Tang, Z., Zhang, L. Depressurization amorphization of single-crystal boron carbide, *Phys. Rev. Lett.* **102**, 075505 (2009).
